# C-Reactive Protein (CRP) levels in neonatal meningitis in England: an analysis of national variations in CRP cut-offs for lumbar puncture

**DOI:** 10.1186/s12887-018-1354-x

**Published:** 2018-12-03

**Authors:** Jonathan P. Sturgeon, Beatrice Zanetti, Dwight Lindo

**Affiliations:** Department of Paediatrics, Kingston Hospital, Kingston, London, KT2 7QB UK

**Keywords:** meningitis, neonatal, CG149, C-reactive protein, CRP, cut-off, neonatal meningitis, lumbar puncture, cerebrospinal fluid

## Abstract

**Background:**

Recent National Institute for Health and Care Excellence (NICE) CG149 guidelines suggest considering performing a lumbar puncture (LP) to investigate for meningitis in early-onset sepsis in a neonate when a C-reactive protein (CRP) level >10mg/L, but the evidence for this recommendation is poorly defined.

**Methods:**

Data on trust-wide LP protocols, neonatal meningitis incidence, lumbar punctures, and CRP levels seen in cases of neonatal meningitis were asked of all 137 trusts in England that recorded a birth in 2017. Our local Kingston Hospital data on every LP performed was obtained to estimate the specificity of CRP rises.

**Results:**

73/123 (59.3%) of trusts follow the NICE CG149 recommendation of considering an LP if the CRP >10mg/L. The national incidence of neonatal meningitis was 0.467/1,000 births, and an LP was performed in 1.37% of all babies, which was significantly higher in trusts considering the CRP > 10mg/L cut-off. A CRP > 10mg/L cut-off sensitivity was 88.9% based on the highest CRP level 4 days around the LP from national data of 199 cases; specificity was 78.8% based on our single-unit analysis.

**Conclusions:**

Proposing a universal CRP > 10mg/L cut-off for a lumbar puncture has been counter-productive in England. Following it generates significantly more LPs, to the point that 40.7% of trusts have chosen not to follow it. It also has poor sensitivity missing over 11% of meningitis. We therefore do not recommend a universal cut-off, rather considering the whole clinical picture (including prematurity) when considering whether to do an LP.

## Background

Neonatal meningitis is a potentially serious infection which occurs in around 0.25 – 1.0 per 1000 live births [1, 2]. Although the case-fatality rate of neonatal meningitis has fallen to around 6.6% for all-cause meningitis [1] or 12.4% for GBS meningitis [3], the morbidity associated with the disease remains unchanged over the last 30 years [2, 4].

A lumbar puncture (LP) is required to investigate for meningitis by sampling cerebrospinal fluid (CSF) for microscopy, bacterial culture, and protein and glucose levels. The decision of when to perform an LP in a neonate can be a difficult one. For those with early onset sepsis (usually <72h old), the American Academy of Pediatrics (AAP) recommend performing an LP if the blood culture is positive, the ‘clinical course or laboratory data strongly suspect bacterial sepsis’, or if the infant does not respond to antimicrobial therapy [5]. In England and Wales, the National Institute for Health and Care Excellence (NICE) have published CG149 guidance suggesting an LP be ‘considered’ if the blood culture is positive, the baby does not respond to antimicrobial therapy, or if the baby has a ‘C-reactive protein concentration of 10 mg/litre or greater’ [6]. This decision to name a specific cut-off CRP of 10mg/L has been criticised by some, with a previous study carried out in 2014 finding that when only the 59 regional referral neonatal units were examined just 14% (10/56) of them followed this CRP guidance [7].

This study therefore aimed to determine the compliance of all neonatal units in England with this NICE CG149 guidance, the number of LPs performed and the number of neonatal meningitis cases seen, as well as the CRP levels seen in these cases in order to estimate a sensitivity for a CRP cut-off of 10mg/L in neonatal meningitis. A recent evaluation of all the lumbar punctures performed at our local unit (Kingston Hospital, London) is also included.

## Methods

### Assessment of national picture of neonatal meningitis for all NHS trusts across England

A list of all 137 NHS trusts at which a birth had taken place in England in the most recent maternity report was obtained from NHS Digital [8]. Requests were made under the Freedom of Information Act (2000).

#### CRP Level for lumbar puncture

Each trust was asked for their neonatal sepsis and lumbar puncture protocol as of 13/2/2018, and whether there was a specific CRP which would warrant a lumbar puncture. Responses were assessed as to whether any ‘consideration’ was given to a lumbar puncture because the CRP was >10mg/L, and whether there were any additional levels stated at which a lumbar puncture should be done. If different numbers for different hospitals in the same trust were given, the highest number was captured.

#### Lumbar puncture and meningitis incidence

For the period between 01/10/2015 (when some ICD10 codes were changed) and 31/3/2018 each trust was asked for the number of episodes with Z38.*, representing any live birth by any mode of delivery, the number of these which also had a lumbar puncture procedure code A55.9, and finally which of these received a G00.*, G01.*, G02.*, or G03.* code for meningitis.

#### Maximal CRP in meningitis

Each trust that had >5 episodes of neonatal meningitis was asked to provide the highest CRP level seen in the 4 days before or 4 days after the positive lumbar puncture for each patient (defined as culture positive, WCC > 20/mm^3^, or PCR positive) – only providing a result if there were a positive CSF.

### Assessment of local lumbar puncture results at Kingston Hospital

Every CSF sample received from the neonatal or post-natal wards at Kingston Hospital by the laboratory between 15/12/2014 – 31/1/2018 was included. Kingston rigidly interpreted the NICE CG149 guidelines, with the trust’s guidelines introduced in June 2013, aiming to perform an LP on every neonate with a CRP > 10mg/L, as well as if any positive culture or clinical concern.

The CSF protein, glucose, cell count, and CSF culture result were noted, as well as the maximal blood CRP in the first three days after starting antibiotics, and blood culture result. The baby’s age and gestation were also noted. A lumbar puncture was considered culture positive if the CSF grew an organism, and ‘cell count’ positive if there were more than 20 WCC/mL in the CSF sample.

### Data analysis

Data were analysed using SPSS (v24, IBM Corp); graphs were prepared using Graphpad Prism (v7.0c, Graphpad software); trusts were mapped using Tableau Desktop (v10.5, Tableau Software). Groups were compared using Mann-Whitney U test with significance being considered at *p*<0.05, having confirmed the data was not normally distributed.

## Results

### National data

Out of the 137 trusts listed in England that recorded a birth in 2016-7, because of three mergers and two trusts only providing community care with no inpatient facilities, at the time of the requests there were 132 trusts that could provide treatment to a potentially sick neonate. All were successfully contacted to request the desired information.

#### CRP level for lumbar puncture

**Fig. 1:**
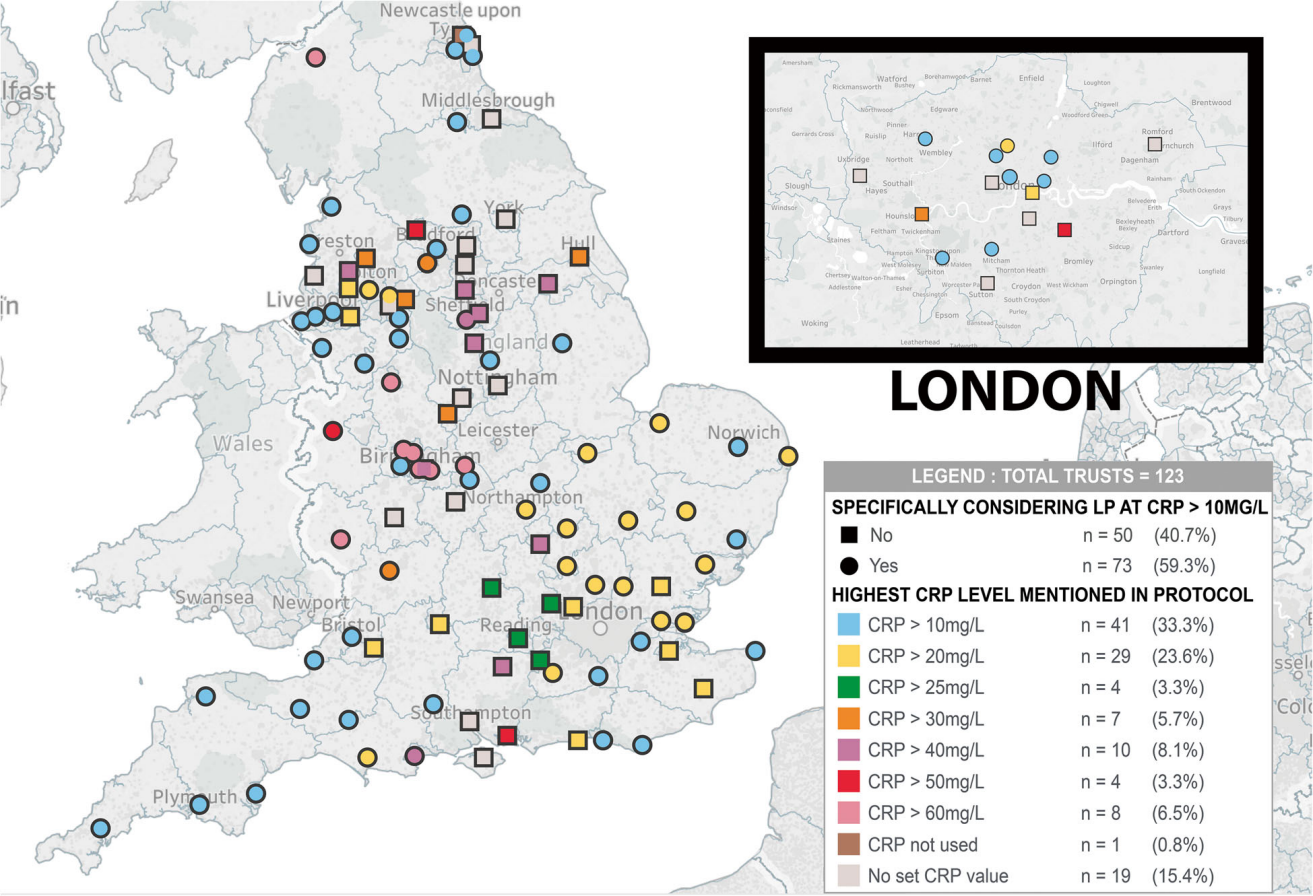
Map of England showing the trusts which consider a neonatal LP at CRP > 10mg/L, as well as the maximum CRP cut-off for an LP mentioned in their protocol. Map background © OpenStreetMap contributors, and is reproduced under the Open Database Licence; information is available from openstreetmap.org

A total of 123/132 (93.2%) of trusts responded. 73/123 (59.3%) of trusts gave some degree of consideration to a lumbar puncture when the CRP was over 10mg/L, therefore directly following the NICE CG149 guidelines. The remainder either did not give any CRP value – leaving the decision for a lumbar puncture up to treating clinician (20/123, 16.3%), or gave a higher CRP level at which lumbar punctures would be considered (30/123, 24.4%, range 20 – 50 mg/L, median 20mg/L). Data shown on the map in Fig. 1*.*

Of the 73 trusts that did give some form of consideration to an LP with a CRP of >10mg/L, 32 gave a further de facto higher level for babies who were asymptomatic, usually on the postnatal ward (range 20 – 60 mg/L, median 20mg/L).

#### Lumbar puncture and meningitis rates

**Fig. 2:**
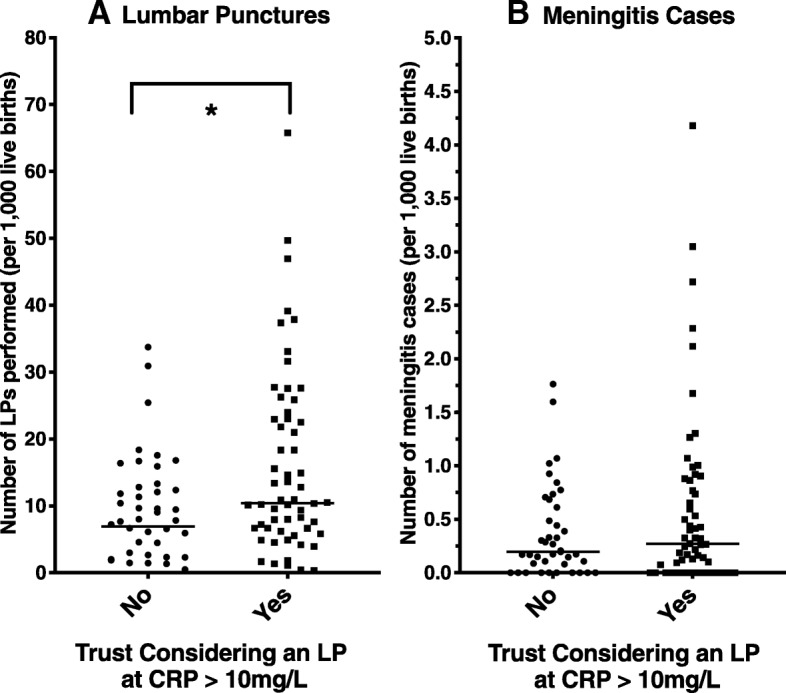
Graphs showing **a** the number of LPs done per 1000 live births by trust and **b** the number of meningitis cases diagnosed per 1000 live births. Each dot represents an individual trust, separated in to trusts which consider a lumbar puncture (LP) at CRP >10mg/L or not. The bars represent the median values. * = *p*<0.05

101/132 trusts (76.5%) provided full data on lumbar punctures and meningitis rates. On top of this, eight trusts provided their number of cases of meningitis in the period as ‘under five’ over concerns over identification of patients, and three trusts inadvertently provided the diagnosis code of A55.9, rather than the procedure code, and did not respond to requests to correct it. These data were excluded from analysis. There were 1,233,485 live births between 1/10/15 and 31/3/18. Of these 16,963 (1.37%) also received a lumbar puncture coding, and 576 also received a meningitis diagnosis in the same episode, representing a nationwide incidence of 0.467/1000 live births. Trusts considering an LP at CRP > 10mg/L carry out a significantly higher number of LPs compared with trusts that do not specifically mention this cut-off (median 1.07%, IQR 0.65 – 2.3%, of live births v 0.85%, IQR 0.34 – 1.31%, *p*=0.042), without a corresponding significant increase in meningitis cases (0.27/1,000 live births, IQR 0.00 – 0.87, v 0.20/1,000, IQR 0.04 – 0.64, *p*=0.54), as shown in Fig. 2.

#### CRP rises in neonates with meningitis

**Fig. 3:**
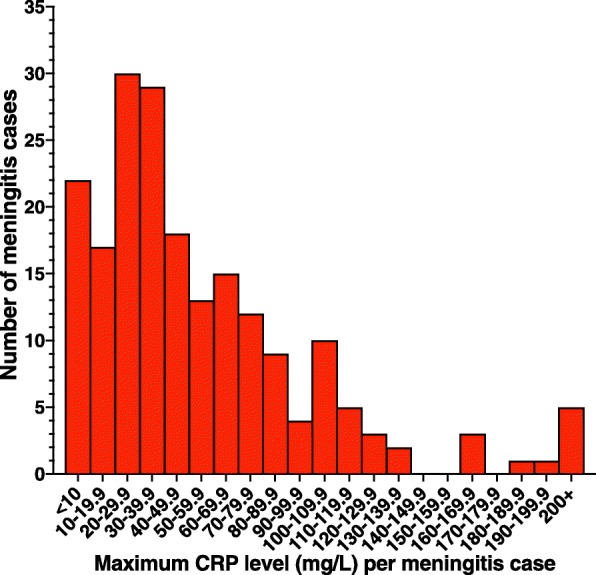
The maximal CRP levels seen in cases of neonatal meningitis, using the national data

CRP levels were provided for 199 cases of neonatal meningitis out of a possible 393 cases, representing a response rate of 50.6% of cases from trusts with over 5 cases. The mean maximum CRP was 56.7mg/L, with 22 cases (11.1%) having a maximum CRP level of <10mg/L in their meningitis infection; this represents a sensitivity of 88.9% for that cut-off in all neonatal meningitis. The breakdown of the maximum CRPs seen is shown in Fig. 3.

### Local (Kingston Hospital) lumbar puncture data

**Fig. 4:**
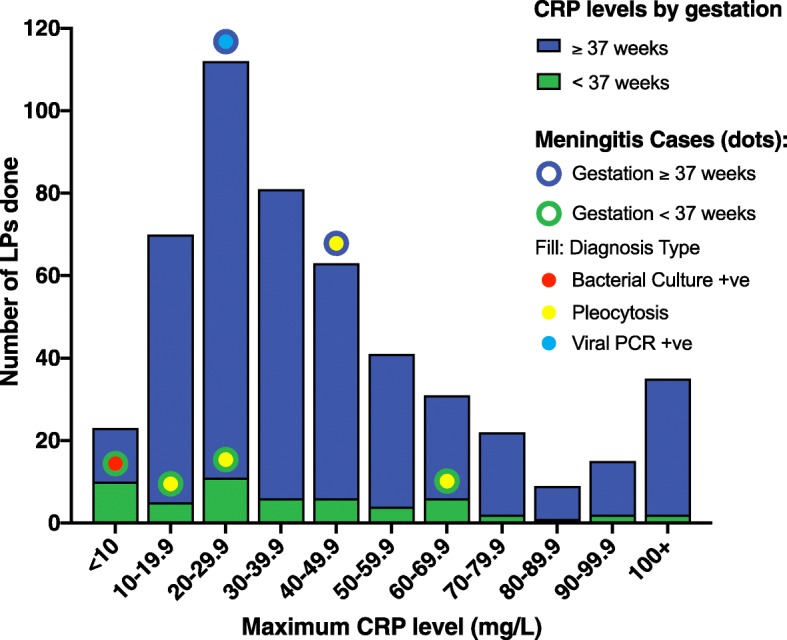
Graph showing the maximal CRP levels seen in those babies who had a lumbar puncture (LP) at Kingston Hospital, along with the cases that had meningitis

511 CSF samples were sent from the neonatal unit or postnatal ward at Kingston Hospital between 15/12/2014 – 31/01/2018. There was an LP frequency of 2.76% of live births. The mean maximal CRP seen in the babies was 36.9mg/L. The breakdown of all LPs by neonatal maximal CRP are shown in Fig. 4.

There were 12 CSF samples that were positive on bacterial culture, 11 of these were commensal organisms and on consideration of the whole clinical picture of the baby the attending physician considered them contaminants. The culture-positive CSF sample was *Enterobacteriaceae* growth in a premature baby who had a maximum CRP of < 10 mg/L, with the LP done because they also grew the same organism in their blood culture. A total of 224 samples were also tested for virology, and 1 was positive with an enterovirus with a maximal CRP of between 20 - 29.9 mg/L.

Four further babies were treated as meningitis because of a significant pleocytosis, with a white cell rise to >20/mm^3^ in the CSF. Two additional cases of mild pleocytosis (20-35/mm^3^) were considered by the attending physician to be because of a traumatic tap, and were not treated as meningitis.

There were 2273 incidences of babies being screened and treated for sepsis, making them potentially eligible for an LP. As no baby with CRP <10mg/L was subsequently re-admitted with a positive CSF, it is reasonable to assume they are likely not to have had meningitis. With this assumption, this represents a local specificity for the CRP >10mg/L cut-off of 78.8%. The local positive predictive value (PPV) of CRP > 10mg/L cut-off was 1.0% overall, but broken down was 0.4% for babies over 37 weeks’ gestation, and 5.6% for pre-term babies.

## Discussion

This study looked at the adoption of the NICE CG149 CRP >10mg/L cut-off for considering an LP in neonatal units in England, the number of LPs and cases of neonatal meningitis diagnosed, the CRP rises seen in these cases, as well as the breakdown of all neonatal LPs performed at our local unit.

The observed neonatal meningitis incidence rate of 0.467/1,000 live births fits well with the 0.25 – 1.0/1,000 values already reported in the literature [2]. The national neonatal LP rate of 1.37% is slightly lower than the 1.7% previously quoted from a single-centred study from 1995, [9] which was before the introduction of the 2012 CG149 guideline, and lower than the Kingston LP incidence of 2.76% which rigidly used a CRP >10mg/L cut-off. This suggests following the release of the CG149 guideline, the number of LPs had not increased, perhaps reflecting the level of its adoption.

Only 59.3% of the 123 local trusts who responded have guidelines that specifically follow the NICE CG149 CRP >10mg/L cut-off for ‘considering’ an LP in a neonate, with the remainder either not having a level, or having a higher cut-off. The CRP level is just one of the three NICE CG149 criteria for consideration of an LP, but because of the low incidence of either positive blood cultures [10] or poor response to antibiotics, it is the criteria that most considerations for an LP would fall under. Several trusts expand in their protocol why their CRP cut-off does not follow the national guidance, with one trust simply putting ‘it has been admitted this number is not evidence based’, another saying it was ‘too low’, and there has been concerns that adopting the CRP cut-off increases the number of LPs done [11]. This is seen in this study in the significantly higher rate of LPs done in trusts who have adopted the CG149 CRP criteria in their protocols.

When looking at their explanation for why they chose a CRP < 10mg/L cut-off for an LP, the NICE guideline development group (GDG) based their CRP > 10mg/L recommendation looking at evidence of CRP rises seen in neonatal sepsis. For a sepsis diagnosis their evidence looked at different CRP cut-offs from 2.5mg/L [12] to 10mg/L [13] for diagnosis of sepsis in babies aged under 12 hours [12] to up to 10 days old [14], therefore making their final recommendation based on the increased likelihood of sepsis rather than specifically meningitis. The GDG were no doubt considering that in bacteraemic infants, the meningitis incidence can be as high as 23% [15, 16].

However, CRP rises do not necessarily mean bacteraemia, and vice-versa. CRP responses in neonatal infection are heterogenous: CRP synthesis is delayed during the early inflammatory response therefore CRP has low sensitivity during early phases of the disease, necessitating repeat measurements [17]. Severity of illness, hypoxia, prematurity, and co-morbidities can all blunt a CRP response [18]. In addition, the CRP can also rise secondary to non-infective conditions including respiratory distress syndrome, meconium aspiration syndrome, stressful delivery, perinatal asphyxia or intraventricular haemorrhage [19] meaning it is poorly specific. Although CRP cannot cross the placenta, maternal pro-inflammatory cytokines such as IL-6 can cross the placenta, potentially inducing CRP production in the neonate [20]. Consequently, studies have shown that maternal fever and prolonged labour can increase CRP in uninfected neonates [21]. These non-infectious rises can be significant – one study of 859 well neonates showed a mean CRP rise to 4.1mg/L by 48 hours, with the 95% centile of the results rising to 13.3mg/L [22].

Because of the low incidence of neonatal meningitis, few studies have been done on the CRP rises in neonatal meningitis, and those done have small numbers: the sensitivity of a CRP > 10mg/L cut-off has been suggested to be 76.3% in one study, [23] and a CRP > 40mg/L to be 72.7% at 24h [24]. This study is therefore able to add a significant body of data representing the CRP rises in neonatal meningitis with a positive CSF (either culture positive, WCC > 20/mm3, or PCR positive), with poor sensitivity and specificity for a CRP > 10mg/L cut-off. Being the highest recorded CRP value 4 days around the positive LP, the timings of the CRPs in this study do not match the initial and 18-24h timings of the NICE CG149 guideline. This wider timeframe was chosen to ensure the highest CRP rise was captured when an LP could have been done at any time during the course of the illness. If the CRP rise could have been restricted to the CG149 initial and 18-24h repeat values of the septic episode, the sensitivity is likely to have been even lower.

Limitations of this study are related to how the data was obtained, through Freedom of Information Act (2000) requests. As well as the administrative burden it places on trusts, no personally identifiable information could be obtained, so information such as the neonate’s gestation could not be requested. Gestation is significant with neonatal sepsis: premature babies below 33 weeks’ gestation, and low-weight infants are more likely to get infections, [1] and their CRP rises are likely to be less. Reflecting the likely different response to meningitis in term babies, several one-centre reports [25–27] as well as one review question, [28] suggest no asymptomatic term baby had meningitis regardless of CRP level. This is reflected in our local results with the CRP > 10mg/L cut-off demonstrating a significantly lower PPV at 0.4% for term babies, but 5.6% for pre-term babies. Finally, because Hospital Episode Statistics were used to identify cases of neonatal meningitis, and each case required a Z38.* birth code given once only, any neonate who had meningitis after discharge or transfer would not be included.

Finally, because there is no coding differentiation between early- and late-onset sepsis meningitis, both are included in this study. Although the organisms causing late-onset sepsis may be different, CRP responses in early- and late- onset sepsis are similar with potentially more sensitivity for sepsis in late-onset sepsis [29].

## Conclusions

With the dissimilar causes and responses of CRP rises in different-gestation neonates, and the lack of evidence of CRP changes in neonatal meningitis, it is easy to see why the AAP did not recommend a laboratory-marker cut-off for doing an LP [5]. The NICE GDG may well have been attempting to ensure all neonates with meningitis were included by selecting a specific CRP for their LP criteria. Recognising these criteria were poorly specific they included the instruction to only ‘consider’ an LP if CRP > 10mg/L or one of the other criteria were filled – allowing for physician discretion to reduce unnecessary LPs. This approach has practically been counter-productive when considering its adoption, on both sensitivity and specificity grounds. Because physician discretion is not conducive to black-and-white instructions on local protocols, 40.7% trusts have not adopted it presumably due to the number of LPs it would require given the test’s low specificity on the background of low-incidence disease. It also fails to include 11.1% of neonatal meningitis cases. Because of both of these failures, it does not seem prudent to assign a universal CRP cut-off for consideration of an LP, rather the decision should be multi-faceted taking in to account the gestation, clinical assessment, microbiology results, strength of concern, as well as any blood results such as CRP.

## Abbreviations

AAP: American Academy of Pediatrics
CRP: C-reactive protein
CSF: cerebrospinal fluid
GDG: guideline development group
LP: lumbar puncture
NICE: National Institute for Health and Care Excellence
PPV: positive predictive value
